# Biobanks, offspring fitness and the influence of developmental plasticity in conservation biology

**DOI:** 10.1590/1984-3143-AR2023-0026

**Published:** 2023-08-28

**Authors:** William Vincent Holt

**Affiliations:** 1 Academic Unit of Reproductive and Developmental Medicine, University of Sheffield, Sheffield, United Kingdom

**Keywords:** biodiversity, extinction, cryopreservation, amphibians, corals, genetic resources

## Abstract

Mitigation of the widely known threats to the world’s biodiversity is difficult, despite the strategies and actions proposed by international agreements such as the United Nations Framework Convention on Climate Change (UNFCCC) and the Convention on Biological Diversity (CBD). Nevertheless, many scientists devote their time and effort to finding and implementing various solutions to the problem. One potential way forward that is gaining popularity involves the establishment of biobank programs aimed at preserving and storing germplasm from threatened species, and then using it to support the future viability and health of threatened populations. This involves developing and using assisted reproductive technologies to achieve their goals. Despite considerable advances in the effectiveness of reproductive technologies, differences between the reproductive behavior and physiology of widely differing taxonomic groups mean that this approach cannot be applied with equal success to many species. Moreover, evidence that epigenetic influences and developmental plasticity, whereby it is now understood that embryonic development, and subsequent health in later life, can be affected by peri-conceptional environmental conditions, is raising the possibility that cryopreservation methods themselves may have to be reviewed and revised when planning the biobanks. Here, I describe the benefits and problems associated with germplasm biobanking across various species, but also offer some realistic assessments of current progress and applications.

## Introduction

The past century has seen the rise of important and severe environmental changes, most of which are attributable to human activities. Well known examples include changes in land and water use, the industrialization of agriculture, fishing and tourism, not to mention global warming and climate change. These influences have had profound, and usually negative, impacts on every aspect of the world’s biodiversity. For up to date and detailed information see the reports of the International Panel on Climate Change (IPCC): (https://www.ipcc.ch/report/sixth-assessment-report-working-group-ii/). Global warming and climate change have far-reaching effects on human and animal populations alike, often facilitating the transmission of viral, bacterial and fungal diseases that affect a multitude of species. Apart from the upsurge in pandemics, such as the ongoing and devastating Covid 19 outbreak across the world, many amphibian species have been decimated by the global spread of the lethal and highly infectious fungal disease, Chytridiomycosis (Rollins-Smith, 2020). A 2004 assessment of global amphibian populations (Stuart et al., 2004) estimated that 34 species had disappeared completely and nearly 2000 were under threat of extinction. Unfortunately, these figures have increased over the last two decades, and the number of amphibian species threatened with extinction is now about 2,500 (out of a total of 7,296, assessed by the International Union for the Conservation of Nature’s “Redlist” (IUCN, 2022). As discussed later in this paper, considerable scientific and technical effort has been invested in rescuing some of these amphibian populations via assisted breeding techniques, and important advances have been made over the past 3 decades.

A recent assessment of global fish diversity (Miranda et al., 2022) estimated that the number of recognised fish species is now over 36,000, of which about 20,000 are regarded as being under some level of threat. Significantly this assessment showed that freshwater fishes are more vulnerable than marine species to the impacts of pollution, much of which is attributable (among other reasons) to contamination from agriculture, mining effluents containing heavy metals, pesticides, chemicals involved in the manufacture of both plastics and cosmetics, as well as the release of human sewage with its content of endocrine disrupting chemicals (Kanda, 2019, Marlatt et al., 2022) and pharmaceuticals (Sanderson et al., 2004). Many of these chemicals are known for their undesirable impacts on embryonic development in wild species and impaired reproductive functions, not only in fishes (Tyler and Jobling, 2008), but also in birds, reptiles, amphibians and many marine crustaceans (Beyer et al., 2022).

As reproductive biologists we may not be able to play a major role in mitigating or reversing the negative impacts of climate change, global warming and industrial practices, but we can try to understand why and how these processes exert their impacts. Moreover, reproductive biotechnologists may be able to contribute their skills to the mitigation efforts, especially through technologies such as cryobiology, the biobanking of reproductive cells and tissues for future use in supporting, or even regenerating, threatened populations (Holt and Comizzoli, 2021). Some of the available technologies have their origins in agriculture and fisheries, where it is now commonplace to use artificial insemination (AI) for breeding domestic mammals and some birds, and to use cryopreserved spermatozoa for breeding over 200 commercially important fish species (Boe et al., 2021; Mayer, 2019). However, it is very clear that for wild species, sperm cryopreservation techniques have to be refined for every target species (Comizzoli and Holt, 2022; Holt and Comizzoli, 2021). The diversity of species, their reproductive behaviors, social systems and respective anatomical peculiarities, is responsible for the technological difficulties that may be encountered when attempting to develop a novel cryopreservation protocol for a conservation-sensitive species. In addition to this caveat, it is now becoming apparent that some reproductive technologies, such as gamete cryopreservation, may inadvertently lead to inadequate post-fertilization offspring growth and survival, while disease resistance may also be suboptimal.

Some of these considerations would have been almost unthinkable as little as 40 years ago, when the possibility that assisted reproductive technologies (ARTs) might cause alterations in the way that genomic DNA sequences are transcribed and translated (i.e., the science of epigenetics) was still unrecognized. The purpose of this review is twofold: on the one hand to outline the undoubted conservation benefits that have, and will be, provided by the use of ARTs, but on the other, to examine the growing evidence that reproductive fitness in some species may be negatively impacted by the laboratory procedures involved.

## Principles of biobanking for conservation

**Figure 1:**
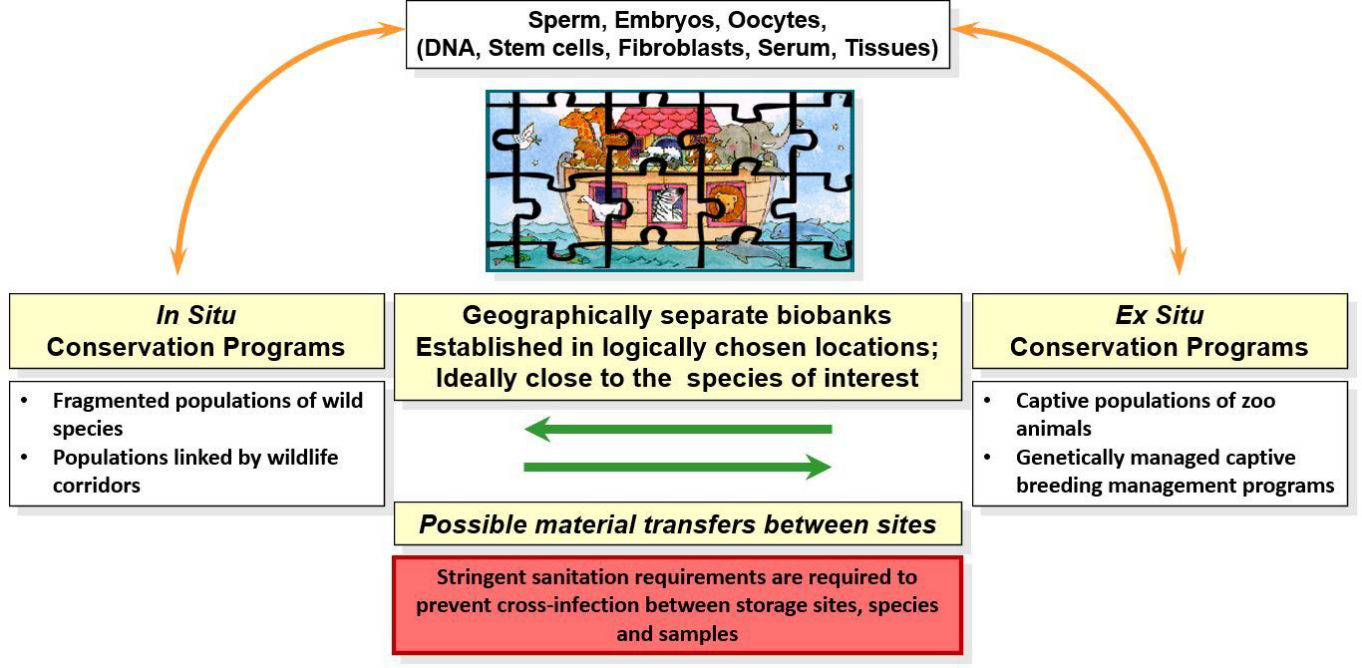
Schematic illustration of the general principles of biobanking for species conservation. Long-term storage of gametes, embryos and other genetic material is accomplished via cryopreservation or other methods such as desiccation. Cell storage takes place at multiple sites in a system of containers for sanitary, geographical and regulatory limitations. Biobanks located in different countries/continents, must comply with stringent international import/export regulations, transportation and political climates that preclude translocations and exchanges. It is also worth noting that storage capability of genetic materials near to the relevant site(s) of eventual use, would represent considerable logistical advantages, by avoiding the expense and difficulty of transporting them by land or air. An additional, advantage of keeping the stored materials close to the relevant wild populations would mean that in the future, when ART techniques for wild species are more reliable and efficient, the biobank may also be considered as a feasible analogue of a natural wildlife corridor, thus permitting isolated and fragmented populations to exchange and share their genetics via the use of advanced technology.

Wildlife conservation actions are undertaken at a wide range of scales. These include projects that aim to improve the biodiversity in a local area such as a lake, woodland or even a small garden, up to the establishment of government-backed national parks or marine protected areas. In some cases, large and small zoos, wildlife parks and aquariums focus their attention on a particular species or group of species, and establish a captive breeding program that aims to minimize the deleterious risks associated with the inbreeding that eventually occurs in small populations (Howell et al., 2021). The captive breeding programs are usually managed carefully, often with different organizations collaborating across international boundaries, in order to ensure the prevention of inbreeding. However, such “live” breeding programs have to operate in a world where animals are not immortal, and it may eventually be impossible to form genetically ideal breeding pairs if one of the breeding animals dies. This situation can be overcome to some extent by establishing a so-called genetic resource bank (GRB) (Holt et al., 1996; Watson and Holt, 2001), or biobank (Comizzoli, 2017; Herrick et al., 2016), which contain stored and preserved germplasm (e.g., spermatozoa, oocytes and embryos), and might still be used for breeding, thus contributing to the genetic aims, long after the animal’s death (See Figure 1).

There are other reasons for attempting to preserve germplasm from threatened species, including capturing genetic snapshots of the dwindling populations before they succumb to extinction. While a database of DNA sequences is undoubtedly useful for evolutionary research, it would miss the diverse associated RNA species that are now known to be functionally important for many aspects of development and survival.

Maintaining a carefully curated collection of viable cells and tissues contributes towards one of the most important aims of conservation biology, namely, to support and augment the ever-disappearing genetic diversity that occurs within endangered populations (Comizzoli, 2017; Holt and Comizzoli, 2021). Viable germplasm in effective long-term storage facilities is expected to remain intact and undamaged for hundreds, if not thousands, of years if maintained appropriately (Whittingham et al., 1977), which means that the genetic properties of gametes and embryos (and stem cells when available) could still be used to counter the deleterious impacts of inbreeding long after the donor animal has died. The use of such conservation-focused biobanks is therefore realistic, provided the appropriate preservation technologies (for example, conventional freezing, vitrification and drying (Anzalone et al., 2018)), and appropriate assisted fertilization methods (including techniques to synchronize the availability of sperm and eggs, and in the case of species with internal fertilization and embryonic development (for example, mammals, birds,, some fishes and reptiles), the ability to synchronize embryonic development with the physiological status of the recipient female reproductive tract. Ensuring that these technologies are all available when required is not trivial, but neither is it impossible. The highly endangered black-footed ferret (*Mustela nigripes*) was rescued from near extinction (Santymire, 2016; Santymire et al., 2014) by intensive and successful research, including the combined use of sperm cryopreservation, biobanking and artificial insemination to restore genetic diversity (Howard et al., 2016).

The financial burden of establishing species and conservation-directed biobanks, in support of associated captive breeding programs, has typically proven to be a strong disincentive for commercial zoos, given that it is not possible to plan for a future time when such support will no longer be needed. In addition to supporting the live, captive populations, of necessity the biobanking costs will include a maintenance requirement for buildings, equipment, electrical power, laboratory reagents and staff. Howell and his colleagues recently published a unique economic cost-benefit analysis in which the financial costs were considered in the context of conserving a particular amphibian species, the Oregon spotted frog (*Rana pretiosa*), for which robust financial data exists (Howell et al., 2021).

The authors of that study made a very strong case for the inclusion of biobanking as a conservation strategy in support of captive breeding programs. It is worth quoting the first paragraph of the discussion section within their paper, which concisely sums up their findings very well.

We present powerful theoretical support for the proposition that biobanking technology should be considered as a complementary tool in captive breeding programs. The use of such technology would address the two most common criticisms of captive breeding: cost and loss of wild-type genetic diversity and fitness. We showed this with models integrating biobanking scenarios into estimates of costs and inbreeding in the captive management of a colony of R. pretiosa. These models demonstrated huge reductions in costs and required sizes of the live colony while achieving reductions in the rate of inbreeding sufficient to achieve and exceed the suggested standard for captive genetic diversity (< 10% loss in heterozygosity after 100 years). (Howell et al., 2021, p. 1).

Although this study was focused on amphibian conservation, the theoretical modelling is more widely applicable and will be of considerable use to biologists justifying similar programs for other taxonomic groups.

The widespread storage, exchange and use of frozen semen and embryos from commercially important mammalian, avian and fish species relies on the observance of strict health and hygiene practices for its success. National and international regulations have been established in order to prevent the inadvertent transmission of diseases (for example, see the detailed regulations that govern terrestrial species from OIE Terrestrial Animal Health Code, 2018). These regulations are based on a detailed understanding of the risks associated with natural and assisted animal breeding practices. However, applying these principles to the biobanking of germplasm from threatened species is typically much more difficult. The risks of disease transmission between species are heightened if the frozen germplasm samples from different species, carrying different and possibly unknown micro-organisms, are maintained within the same liquid nitrogen container (Bielanski, 2014). This complicates, and probably precludes, the popular concept of a cryopreservation container filled with frozen samples from a range of different species. The establishment of biobanks in support of particular species or populations is nevertheless entirely feasible if samples are prepared and stored as near as possible to the population requiring genetic support. In essence this means that, where possible, it is best to avoid transporting samples across international borders and fulfilling the sometimes onerous and complex import and export requirements imposed by animal health authorities.

An emerging issue that is likely to gain more importance in the future concerns the use of animal-derived products such as egg yolk, skimmed milk and bovine serum albumin, in cryopreservation media. The regulatory frameworks governing the international movement of germplasm for agriculture and fisheries require assurances that these products are not themselves infected with transmissible disease vectors. As biobanking becomes more widely applied across a range of wild species, practitioners will need to remain mindful of these risks and avoid them where possible by finding alternative, possibly synthetic, additives. To date this search is still ongoing as the reliability of substitute preparations is usually questionable (for further information see references (Akhter et al., 2021; Kucuk et al., 2021; Li et al., 2005; Sicchieri et al., 2021).

## What is the current status of mammalian biobanking for conservation?

Currently, two well-organized wildlife biobanks containing frozen semen have contributed to the genetic support of threatened wild animal populations; as discussed above; one is focused on the black-footed ferret recovery program, and the other supports the Giant panda breeding program in China.

The Giant panda breeding program in China has amassed sufficient data to examine success rates of AI with frozen semen and to compare them with natural breeding outcomes (Li et al., 2017). An analysis published in 2012 found that the success rate of AI with frozen semen up to the year 2011 was about 25%; (5/20 AI events with cryopreserved semen resulted in live offspring). This is slightly higher than a later estimate (18.5%) published in 2017 (Li et al., 2017), which was based on 65 AI events, carried out using both fresh and cryopreserved semen, although lower than the birth rate of 60.7% obtained after natural mating. Successful use of frozen semen for the giant panda AI has also been achieved outside China (Comizzoli, 2020).

Interestingly, the factors determining AI success in the giant panda (Huang et al., 2012; Li et al., 2017, Martin-Wintle et al., 2019; Spindler et al., 2006) have revealed that the quality of frozen semen is less important than the timing of the insemination itself.

As with many other mammals, matching the timing of insemination and the occurrence of ovulation requires precise information about ovarian status. The lifespan of frozen-thawed semen within the female reproductive tract may be very short (possibly less than 4 hours in some species, and therefore if ovulation occurs 5 hours or more after insemination, fertilization would not take place. While information about ovulation status can be obtained using ultrasound scanning in readily handled domestic species, this is not practical in most wild species that, of necessity, require sedation or anesthesia for such examinations (See, for example, a recent study of brown bear ovarian dynamics (Torii et al., 2020). Successful AI in the giant panda is known to depend on very precise estimations of ovarian status, either by the regular examination of cellular morphology in vaginal smears (Durrant et al., 1998) or the regular (8-hourly intervals) measurement of urinary oestrogen, progesterone and luteinizing hormone (Martin-Wintle et al., 2019). Greatest success using the hormonal measurement method was obtained when AI was performed within 40 hours of the decline in urinary oestrogen concentration.

A number of European zoos have supported an EAZA (European Association of Zoos and Aquariums) initiative to ensure that the genetic diversity of wild felid species is protected and preserved (Fernandez-Gonzalez et al., 2019) in a dedicated biobank. Male and female gonads from 74 females and 67 males, from 36 different zoological institutions, have been donated to the project, which is organized by the Leibniz Institute for Zoo & Wildlife Research in Berlin, Germany, whose reproductive biology department has a specific focus on biobanking for conservation (https://www.izw-berlin.de/en/biobanking-for-assisted-reproduction-techniques.html.). The 2019 publication (Fernandez-Gonzalez et al., 2019) explains that epididymal spermatozoa have been collected from 42 males (63%) and that samples from 36 males (54%) were suitable for freezing. This project has used the spermatozoa and oocytes to generate 47 felid embryos by oocyte maturation and *in vitro* fertilization, including one Asiatic golden cat embryo and 8 from two Northern Chinese leopards. As this is an ongoing project, and can be regarded as an active biobank, these numbers are expected to increase as more zoos donate samples.

Research progress leading towards the establishment of biobanks for other mammalian species is still at an early stage, possibly because of the technical difficulties associated with effective sperm cryopreservation despite extensive research efforts (see, for example, the difficult problems around cryopreserving marsupial spermatozoa (Johnston, 2019; Johnston and Holt, 2019). Some researchers have argued that because sperm cryopreservation is so unlikely to succeed by the use of traditional cooling and freezing methods, it is now time to develop alternative approaches to genetic biobanking based on the use of cloning and stem cell technologies (Rodger, 2019). It is at least theoretically possible to derive gametes from induced pluripotent stem cells (iPSCs), which could then be used for the production of embryos from a range of somatic cells. (Dicks et al., 2021; Stanton et al., 2019; Sukparangsi et al., 2022). Some biobanks, see for example, a project in Thailand aimed at the development of a biobank specifically to support fishing cat (*Prionailurus viverrinus*) conservation (Sukparangsi et al., 2022), are employing this approach as their primary option and the same principles are being explored in relation to the conservation of some aquatic species (Rivers et al., 2020; Temkin and Spyropoulos, 2014). It will be instructive to see whether mammalian offspring that are generated by the exploitation of stem cell technologies show comparable viability and survival as those produced more conventionally. There is currently too little evidence to make informed predictions around this topic, but a number of authors have pointed out the risks associated with epigenetic reprogramming and development (Borges and Pereira, 2019; Wang et al., 2020). Cloning itself, or somatic cell nuclear transfer (SCNT), was popularly expected to revolutionize and facilitate the breeding of endangered species, using the frozen somatic cells contained in biobanks as nuclear donors. However, this approach has become less popular because of problems with the requisite nuclear reprogramming, as well as matching the species of the available nuclear donor with oocytes of the same species. This has led to high levels of embryonic death, placental incompatibility and perinatal mortality (Borges and Pereira, 2019; Dicks et al., 2021; Mastromonaco et al., 2014; Niemann, 2016).

## Biobanks, conservation and the significance of developmental plasticity

The evolutionary literature is replete with examples of species where both males and females have evolved and adapted their physiological, behavioral, and morphological characteristics, optimizing their survival, reproductive success and fitness over centuries and millennia. However, while many of these long-term adaptations have been effected via gene mutation and various kinds of evolutionary selection, many such adaptations are induced by changes in the diverse ways in which gene expression is controlled. Such changes, caused via developmental plasticity, need not be solely determined by the precise nature of the DNA sequences involved. In fact, changing the way that the DNA is expressed permits species to respond quickly to environmental changes. The relevant control of gene expression during development is effected via processes such as DNA methylation, histone and microRNA modifications. These developmental processes have become especially sophisticated in social insects, where they control the behavior, morphology and social roles of different insect castes, all of which develop from eggs produced by a single female (known colloquially as “the Queen”) for review, see (Oldroyd and Yagound, 2021).

Given the enormous influence that epigenetic changes can induce during development, without the necessity of gene mutations, it is worth asking whether the technical processes needed for gamete and embryo cryopreservation, even including the nature of incubation media, might have the capacity to induce functionally significant epigenetic changes that might affect the future survival of offspring. This is not impossible, even though the cryopreservation of spermatozoa has been used successfully in agriculture and human medicine for more than five decades.

The application of ARTs in various human infertility treatments is estimated to have resulted in the birth of over million children in Europe (Schroeder et al., 2022; Wyns et al., 2021). Evidence suggests that a small proportion of the children born following *in vitro* fertilization and embryo transfer suffer from genomic imprinting diseases (Beckwith-Wiedemann, Angelman, Prader-Willi and Silver-Russell syndromes), slightly elevated risk of infant mortality in the first year of life, exhibit signs of large size for gestational age, high birthweight and other problems (for reviews, see Mani et al., 2020; Reyes Palomares and Rodriguez-Wallberg, 2022; Wyns et al., 2021). While some of these problems may be related to the original causes of the infertility, the various technologies used in clinical ART have also been implicated. The situation with agriculturally important animals undoubtedly differs from that with humans, conservation-significant wild animals and farm animals. While agriculturally important animals are subjected to continuous and commercially-driven selection pressures, these do not apply to humans or threatened species. In fact, conservation biologists strive to conserve as much genetic variability and thereby avoid selection processes as far as possible. This means that breeders and farmers would not usually maintain animals that show poor performance characteristics (e.g., milk yield, semen quality, feed conversion efficiency, etc.), which therefore may not be recognized and studied. Nevertheless, one of the first negative impacts of ART to be reported, was the recognition of Large Offspring Syndrome in cattle and sheep (Young et al., 1998); many other aspects of genetic inheritance in agricultural species have been reviewed by Thompson et al. (2020).

Such considerations also raise valid questions about the eventual re-integration of preserved and stored cells into conservation-focused species support and recovery programmes. If spermatozoa, oocytes or embryos have been preserved by cryopreservation, desiccation or other means, and stored carefully for half a century, is there any likelihood that ART applications would fail for technical reasons? This question was investigated about 40 years ago (Whittingham et al., 1977), when frozen mouse embryos maintained at ─196 ◦C were exposed experimentally to background radiation that simulated the equivalent of several thousand years. Fortunately, no deleterious impacts were detected. Investigating the potential occurrence of technically-induced genetic mismatches between genetic materials in long-term storage and their use in contemporary breeding programmes undoubtedly requires other approaches. One interesting possibility involves comparing the epigenetic signatures obtained from contemporary species of conservation interest with historical genomes derived from museum specimens; such studies are currently being carried out with black-footed ferret genomes (Personal Communication: Dr Klaus-peter Koepfli, Smithsonian Conservation Biology Institute, Washington, DC.).

## Biobanking and the genetic rescue of threatened amphibians

The world’s amphibian populations have been in decline over the last few decades, largely owing to the transmission of several infectious diseases (Daszak et al., 1999; DiRenzo and Campbell Grant, 2019), including, among others, both a fungal (Chytridiomycosis) and a viral (Ranavirus) infection (Rae and Murray, 2019; Sutton et al., 2014). These infectious diseases have been responsible for the diminution, or even complete disappearance, of amphibian populations from diverse habitats across the world. However, in turn, this problem has stimulated considerable research effort aimed at either eradicating the disease vector (*Batrachochytrium dendrobatidis* (Bd) (Sigafus et al., 2014), or finding ways that support the wild amphibian populations, and possibly permit them to co-exist with the disease (DiRenzo et al., 2018; Scheele et al., 2019). Current evidence suggests that co-existence is a possibility, given the remarkable developmental plasticity of amphibians and their ability to adapt and survive under diverse, and adverse, conditions (Jonsson et al., 2022; Sun et al., 2020).

The amphibian extinction crisis has also stimulated reproductive biologists to investigate the feasibility of cryopreserving amphibian spermatozoa, storing them in cryobanks and producing offspring that could be used in support of dwindling populations (Browne et al., 2011; Clulow et al., 2014; Kouba et al., 2013; Silla and Byrne, 2019; Uteshev et al., 2019). Besides developing appropriate sperm cryopreservation methods, researchers have developed a range of hormonal treatments for the controlled and reliable collection of male and female gametes for use in *in vitro* fertilization (summarized by Clulow et al., 2019). It is worth mentioning here that, because the amphibian male reproductive tract releases spermatozoa into a cloaca, the spermatozoa are released together with urine. Moreover, the collected spermic urine can be cryopreserved, stored and subsequently used to fertilize eggs (Kouba et al., 2012; Uteshev et al., 2012).

Unlike the situation with wild, but endangered, mammals and birds, a body of preliminary evidence about the development and survival of tadpoles and adults derived from frozen-thawed spermatozoa, is accumulating. In some cases, there is no suspicion that the use of cryopreserved amphibian spermatozoa might result in offspring with compromised survival or fertility (Lampert et al., 2022). Indeed, the successful use of cryopreserved spermatozoa in breeding programs for threatened amphibian species, and for establishing biobanks and insurance populations as a hedge against extinction, is now widely regarded as an important aspect of amphibian conservation (Kouba et al., 2013; Silla and Byrne, 2019; Upton et al., 2021). However, a detailed developmental study of Fowler’s toad reproductive success (Poo et al., 2022) showed that cryo-derived tadpoles and post-metamorphic toadlets were consistently smaller than their naturally-derived counterparts at the same stage. Interestingly, the authors did not detect any negative impacts on post-metamorphosis survival. These results mirror a study which found that the post-hatching growth of wild brown trout (*Salmo trutta)* (Nusbaumer et al., 2019) derived from cryopreserved spermatozoa was significantly lower than control embryos derived from fresh spermatozoa. At present there is no explanation for this effect, although one explanation put forward by Nusbaumer et al. (2019) suggested that these effects might be easily overlooked. It is also worth considering that the effects of amphibian sperm cryopreservation could vary between species, and might even show evidence of heritability. Although there are now a number of other studies in which phenotypically normal embryos were produced by artificial fertilization with cryopreserved frog and toad spermatozoa, and where metamorphosis resulted in normal adults (i.e. (*Xenopus laevis* (Pearl et al., 2017) and Golden Bell frog (*Litoria aurea*) (Upton et al., 2021)), it seems that more research into the heritability question is needed.

## Biobanking for the restoration and support of coral reefs

Considerable attention has recently been focused on the establishment of successful cryopreservation technologies for coral species (Daly et al., 2018; Grosso-Becerra et al., 2021; Hagedorn et al., 2012, 2019) and a number of focused gene banks have already been established around the world (Caribbean, Hawaii, French Polynesia and the Great Barrier Reef; (Hagedorn et al., 2019). Spermatozoa from 31 different coral species have been cryopreserved successfully using a standard technique involving dimethylsulfoxide as the cryoprotectant, and a novel technique for the successful cryopreservation of coral larvae has been developed (Daly et al., 2018). Compared with other organisms there is an added complication to the cryopreservation of coral cells in that many co-exist with dinoflagellate symbionts (see Jiang et al., 2021) for review), whose biochemical functions are also being compromised by climate change, toxic chemicals and other environmental problems. This presents a multidimensional problem, which is nevertheless being addressed successfully.

## Integration of biobanks and biodiversity conservation

Successful sperm cryopreservation has now been achieved in many wild species (Prieto et al., 2014) but success has, in many cases, been recognised mainly by the recovery of cellular structure and function (i.e., motility, plasma membrane and acrosomal integrity and, more recently, the retention of functional mitochondria and undamaged DNA (Paoli et al., 2019; Pollock et al., 2018). While these parameters are informative, they do not necessarily guarantee that spermatozoa will have the capacity to both fertilize oocytes and support appropriate embryonic development. It is therefore pointless to invest in major biobanking projects that rely entirely on the use of cryopreserved, but untested, sperm samples. However, it is worth mentioning that the IUCN Species Survival Commission recently established an Animal Biobanking Specialist Group in 2022, thus endorsing the value that is currently being placed on biobanking as a further resource for species recovery programs (https://www.iucn.org/our-union/commissions/group/iucn-ssc-animal-biobanking-conservation-specialist-group)

Agricultural and fisheries biobanks that focus on commercially significant species and breeds (Blackburn, 2018; Boe et al., 2021), as well as those focused on basic sciences and that store particular genetic lines of mice (Kaneko and Serikawa, 2012; Landel, 2005) and small fishes (Tiersch et al., 2011; Yang and Tiersch, 2009) ensure that their stocks of spermatozoa are reliably fertile. This level of assurance is not currently possible with most wild species, and it is for that reason that I regard only the Black-footed ferret and Giant panda projects as possessing well-established sperm biobanks. It is also true that, despite the current enthusiasm for banking somatic cells in the expectation that they will be used for the production of iPSC cells, artificial gametes and hence embryos (Pessôa et al., 2019), these expectations have yet to be convincingly demonstrated. Biobanks that aim to support the genetic diversity of threatened populations should ideally contain as much of the existing gene pool as practical. This means storing sufficiently large collections of somatic cells to capture the maximum possible genetic diversity (Herrick, 2019; Herrick et al., 2016) and using them to produce genetically diverse offspring that represent the founder populations. Very small numbers of individual animals have so far been produced by this method (Stanton et al., 2019) and, as such, are not yet able to contribute much genetic diversity to existing populations.

Studies of the relationships between climate change and species survival have detected that many species, including birds, amphibians, reptiles, mammals and fishes (Hermes et al., 2018; MacNeil et al., 2010; Morris and Dupuch, 2012; Tiberti et al., 2021) are responding by either going locally extinct (Albano et al., 2021), or by shifting their preferred habitats towards cooler, polar and higher altitude regions. This undoubtedly imposes different selection pressures and disease risks upon populations experiencing those shifts. One relevant, and possibly surprising prediction anticipates that by 2070, 35% of mammals and 29% of birds will have over half of their 2070 climatic niche in countries where they are not currently present (Titley et al., 2021). The organization of biobanks may have to take these predictions into consideration, especially if the relevant germplasm samples intended for the provision of genetic support unexpectedly turn out to have become more suitable for supporting populations in a foreign country. Interestingly, some authors have expressed the view that if an endangered species is already unable to cope with the negative impacts of climate change, translocating the population elsewhere may help it to survive (Schwartz and Martin, 2013; Thomas, 2011) if a suitably hospitable area could be provided.

## Conclusions

The concept of biobanking for conservation is not new, and indeed detailed proposals for a variety of biobanks targeted at different species, were published in the late 1970s (Veprintsev and Rott, 1979). In the 50 years that have elapsed since those early proposals, millions of biological samples, including cells of human, animal or bacterial origin, viruses, serum/plasma or DNA/RNA, are maintained in well-organized international collections. Unfortunately, the technology for exploiting these biobanks for use with wild animal conservation and breeding has been less effective than it might have been, given better financial support. To some extent, this shortcoming can be attributed to conservation biologists themselves, who have frequently dismissed, or ignored, the possible supportive role of cryopreserved biomaterials. This is in sharp contrast to the attitudes of the botanical community, who have been able to develop well-funded, long-term projects for the establishment of seed banks. (See, for example, The UK Millennium Seed bank at Kew in London in the United Kingdom (Chapman et al., 2018; Liu et al., 2018), which now contains seeds from nearly all native UK plants and many from outside the UK).

Over the last 20 years there have been many developments in reproductive technology, driven to a great extent by the requirements of agriculture, aquaculture and human reproductive medicine. The possibility that mammals, such as camels, horses and even sheep, could be cloned (Mastromonaco et al., 2014; Wani et al., 2017), or that fishes of one species can be engineered to produce the gametes of another (Morita et al., 2012), were matters for science fiction. As I have pointed out, there are some question marks over the health and fitness of the offspring that result from these techniques. Nevertheless, it is highly likely that those obstacles will eventually be overcome in time to save at least some of the world’s most endangered species.
